# Aflatoxin B1 Contamination in Chicken Livers and Gizzards from Industrial and Small Abattoirs, Measured by ELISA Technique in Maputo, Mozambique

**DOI:** 10.3390/ijerph14090951

**Published:** 2017-08-23

**Authors:** Alberto Romão Sineque, Custódia Lina Macuamule, Filomena Rosa Dos Anjos

**Affiliations:** 1 Biological Science Department, Science Faculty, Eduardo Mondlane University, Maputo 257, Mozambique; 2 Paraclinic Department, Veterinary Faculty, Eduardo Mondlane University, Maputo 257, Mozambique; custodiamucavele@hotmail.com; 3 Animal Production Department, Veterinary Faculty, Eduardo Mondlane University, Maputo 257, Mozambique; anjosmena@gmail.com

**Keywords:** poultry production, broilers, giblets, aflatoxins, Maputo

## Abstract

Aflatoxins are the most toxic and carcinogenic mycotoxins produced by *Aspergillus* species. Aflatoxin B1 (AFB1) contamination in industrial and local chicken livers and gizzards in Maputo was investigated. One hundred boiler livers and 80 boiler gizzards were collected from industrial and local cutting poultry production sectors. The samples were analyzed by the ELISA method (MaxSignal^®^, Bioo Scientific Corporation). AFB1 was found in 39% of liver samples and 13.8% of gizzards, with mean levels of 1.73 µg/kg and 1.07 µg/kg, respectively. The frequency of contamination and AFB1 levels in samples from local sector producers was not significantly higher than those from industrial sector producers (*p* > 0.05). No correlation was found (*p* = 0.493; *r*^2^ = 0.013) between AFB1 levels in livers and hepatic weights. The AFB1 levels were lower than the allowed limits, suggesting that these products do not pose high risk to consumers. Notwithstanding, there is a need to implement aflatoxin residue monitoring and controls in all chicken meat products; this economic and efficient technique appears to be valuable for improved food safety in Mozambique.

## 1. Introduction

Poultry industry represents an activity of great importance worldwide, including Mozambique, since it constitutes one of the main sources of animal protein available to the population [1]. However, many crops used as main poultry feed ingredients—such as corn, peanut meal, cottonseed meal, and sorghum—are susceptible to mycotoxin contamination, representing a greater risk for introduction of mycotoxins in poultry diets [2,3,4,5].

Feed contaminated with mycotoxins, especially aflatoxins, is often a health and production hazard for poultry. Moreover, mycotoxin residues in poultry products may represent a threat to humans through their carcinogenic, mutagenic, teratogenic, immunosuppressive, and other adverse effects [2,6,7,8]. In addition, annually, many of the mentioned crop and approximately 25% of the world’s food supply are contaminated with mycotoxins [3,5,6,7,8,9].

Mycotoxins are toxic metabolites produced by molds under specific conditions. Among them, aflatoxins produced by toxigenic fungi—mainly *Aspergillusflavus*, *Aspergillusparasiticus*, and *Aspergillusnomius*—constitute some of the most important environmental toxicants which represent a health hazards both for humans and animals [2,6,7,10]. Among the aflatoxins, four major groups—aflatoxin B1, B2, G1 and G2—are emphasized [10,11], with aflatoxin B1 (AFB1),the most toxic and a known carcinogen [10,11,12], being included in Group 1 of carcinogenic agents by the International Agency for Research on Cancer(IARC) [2,5,8,13].

When ingested by animals, including humans, AFB1 is metabolized in the liver by specific cytochrome P450 enzymes into various isomers, including aflatoxin-8,9-epoxide, which may then bind to proteins and cause acute toxicity (aflatoxicosis) [10,14,15,16,17,18] or to DNA and induce liver cancer [2,8,10,18]. In addition to reactive oxygen species, AFB1 metabolism results in the production of aflatoxin M1 (AFM1), which has similar toxic properties to AFB1 [5,8,10,18].

Besides the toxic effects, consumption of AFB1 contaminated feeds by poultry may lead to significant economic losses due todecreases in growth performance and meat quality, poor feed utilization [2,10,18,19,20], and an increase in the incidence of disease in poultry [2,10,17]. This considerable sensitivity of poultry species to AFB1 may be associated with their livers’ efficient capacity to convert AFB1 to the metabolically active aflatoxin aflatoxin-8,9-epoxide [5,10,17,18,20]. However, it is reported that the susceptibility to aflatoxins differs among poultry species [2,5,12,20], with ducks beingthe most susceptible, followed by turkeys, boilers, and laying hens [2,5,17].

Aflatoxin residues, especially AFB1 and its metabolites, may be present in the tissues, milk, and eggs of animals fed with AFB1-contaminated diets to become a potential human health hazard [2,5,7,10,18,21]. It has been demonstrated that AFB1 intake is associated with a high incidence of human liver cancer [3,8,12], and also with the incidence of breast, prostate, and gastrointestinal cancers; protein-energy malnutrition in children; as well as linked with the progression of HIV infection, especially in low-income countries [3,8,22,23]. Since it is considered a major risk to public health, human exposure to AFB1 through animal sourcefood has been reported by several investigators [3,8,9,19,24] and is continuously monitored in developed countries through different chromatographic and immune-enzymatic methods [5,7,25,26,27,28,29,30,31]. This monitoring does not occur in many developing countries, including Mozambique.

In Mozambique, the consumption of chicken meat tends to increase each year. According to FAO [1], in 2011, the total volume chicken meat consumed was 46,572 tons, with an average availability of 1.94 kg of poultry meat per capita. Nevertheless, it was also estimated that 13 percent of the consumed meat was imported and 31 percent of the total domestic production was from the local sector or smallholder family sector [1].

Previous studies have indicated the presence of aflatoxin(s) in poultry feed [32], and also concentrations in main ingredients [33,34] as being above the maximum allowed level by the Codex Alimentarius regulations. There are different regulatory limits for aflatoxin in foodstuffs throughout the world, varying form one country to another [6,24,35,36]. Many developing countries, including Mozambique, have no legal limits for aflatoxins, as a result of economic considerations [6,9,35,36]. Those countries have generally adopted the Codex Alimentarius regulations, which prescribe that the maximum level of AFB1 in human food should not exceed 10 μg/kg (ppb) [6,24,35]. Moreover, in Brazil for example, which is the main source of imported chicken meat in Mozambique, the maximum tolerated level of AFB1 in human food is 5 μg/kg [6].

Considering the above facts and the very limited studies conducted in Mozambique on the occurrence of aflatoxins in chicken meat, the present study aimed to investigate AFB1 contamination in livers and gizzards of industrial and local produced boilers in the Maputo region, using an ELISA quantification method. The results of the present study will be helpful to create awareness of the health hazards associated with these toxins, and also to assist research and monitoring programs to implement regulation.

## 2. Materials and Methods

### 2.1. Sampling

A total of 180 samples (100 liver and 80 gizzards, considered approved for commercialization) were collected randomly between May and June 2016 from four different locations of chicken production and slaughtering facilities in Maputo, Mozambique. Sampling locations included two from the industrial sector or formal producers (IS) and three from the local sector (smallholder family sector) or informal producers (LS). The collected samples were placed into sterile plastic bags (Ziploc type), identified, and transported to the Laboratory of Chemistry, Animal Sciences Department of the, Mozambique Institute of Agricultural Research (IIAM), under refrigerated conditions in an ice box and stored between −4 °C and 0 °C [5,7], until further analysis.

### 2.2. Morphological Evaluation of Liver Samples

During collection, liver samples were weighed using a non-analytical digital balance (1–3000 g) and examined for color, categorizing them as “normal”, “pale or yellow”, and “moderate” livers, according to procedures described in Dos Anjos et al. [16] and the USDA [37]. The “normal” livers were defined as those with range in color from tan to deep mahogany red, while ‘moderate’ livers encompassed those with up to two-thirds of the total area being pale or yellow in color.

### 2.3. Analytical Procedures

#### 2.3.1. Extraction of Aflatoxin B1

Before analysis, equipment and materials used were washed with detergent and distilled water, then, when applicable, sterilized with an autoclave. Extraction of AFB1 from liver and gizzard samples was performed according to the recommendations of the ELISA kit manufacturer. Two (2) grams of individually ground and homogenized samples were blended with 8 mL of 87.5% methanol in a 15 mL centrifuge tube and shaken vigorously for 3 min on vortex. Subsequently, the samples were centrifuged for 10 min at 4000× *g* at room temperature. The supernatant (300 μL) from each sample was diluted with 900 μL of a mixture of 100% methanol and 1× extraction buffer and shaken for 1 min on vortex. In addition, to assess the accuracy of the ELISA measurements [38], two negative liver samples of a preliminary testing were spiked with 5 and 10 μg/kg AFB1.

#### 2.3.2. Analysis of AflatoxinB1 in Samples

AFB1 content analysis was performed by a competitive ELISA method, using the AFB1 MaxSignal^®^ commercial kit (1055-04, MaxSignal^®^, Bioo Scientific Corporation, Austin, TX, USA), which contains 96-well micro-titer plates sensitized with monoclonal antibody specific for AFB1. Fifty (50) μL of each standard solution and each sample, including those artificially contaminated (spiked), were added in duplicate to the wells of the micro-titer plate. Subsequently, 100 μL of aflatoxin B1-horseradish peroxidase conjugate was added to each well of the plate, the plate was manually shaken for 1 min and incubated at room temperature for 30 min. After incubation, micro-titer plate wells were completely emptied and washed three times with 250 μL of the 1× wash solution in each wash, and dried by tapping several times on a paper towel layer. Unbound conjugate was removed during washing. After the washing step, 100 μL of tetramethylbenzidine (TMB) substrate was added to each well of the plate, and the plate was manually shaken again for 1 min and incubated at room temperature for 15 min (counted from the first addition of the substrate). The reaction was stopped by adding 100 μL of the enzyme reaction inhibition buffer, and the absorbance was immediately measured at 450 nm in a BioTek^®^ ELISA plate reader (EL-800, BioTek^®^, Winooski, VT, USA). The absorption intensity was found to be inversely proportional to AFB1 concentration in the sample. AFB1 concentrations, as well as standard curve determination, were processed on a specific aflatoxin MaxSignal^®^ Excel analysis program (Bioo Scientific Corporation, Austin, TX, USA), considering a dilution factor = 20, as recommended in the kit procedures. Besides to the sample spiking’s, an intra- and inter-assay coefficient of variation (%CV) less than 20% [38], as well as a repetition of two positive samples for each assay run were considered as criteria to ensure the required quality (for validation) of the measurement results.

### 2.4. Statistical Analysis

The data were statistically analyzed using Prism software (GraphPad Software, Inc. 5.1, San Diego, CA, USA), and expressed as frequencies or means. Frequency of AFB1 contamination was analyzed using the Chi-square test. AFB1 concentrations were analyzed using analysis of variance (ANOVA) and Tukey multiple test. Correlation between AFB1 levels in livers and liver weights was also analyzed by linear regression. The level of significance was set at *p* < 0.05 for all statistical analyses tests. Recovery rates of artificially contaminated (spiked) samples were calculated by dividing the recovery AFB1 levels with the AFB1 spiking levels.

## 3. Results

In the present study, 100 samples of chicken liver and 80 of gizzard were tested for the frequency of AFB1 contamination using an ELISA technique. In the morphological evaluation, most of the liver samples were found to be normal (47%) or moderate (40%) in color, whereas the weight was higher in livers from local sector (LS) producers with (42.4 ± 5.24 g) compared to industrial sector (IS) producers (38.0 ± 5.48 g); *p* < 0.05 (Table 1). Differences of liver weights between the liver type were also found (*p* < 0.05 ANOVA test), with high weights in pale/yellow (45.59 ± 2.06) and moderate livers (46.03 ± 0.90), for IS and LS producers, respectively.

For the ELISA assays, known amounts of AFB1 were added in two liver samples to determine the recovery rates. These varied between 91% and 93% for 5 and 10 μg/kg AFB1 spiked levels, respectively (Table 2). Frequency of contamination and AFB1 levels in analyzed samples are shown in Table 3; 39% of liver samples and 13.8% of gizzards were found positive for AFB1 contamination. The contamination and AFB1 levels in samples from LS producers were not significantly higher than those from IS producers (*p* > 0.05). Numerically, in the LS, AFB1 was found in 66.7% of livers and 30% of gizzards, in comparison to the IS, which resulted in 27.1% and 4.0% contamination in livers and gizzards, respectively. The highest levels of AFB1 were found in liver samples and the lowest levels in gizzard samples, both from LS producers, averaging 1.73 μg/kg and 1.04 μg/kg, respectively. However, all estimated AFB1 contamination levels were lower than the allowable limit (10 μg/kg) for total aflatoxin in food in Mozambique.

**Figure 1:**
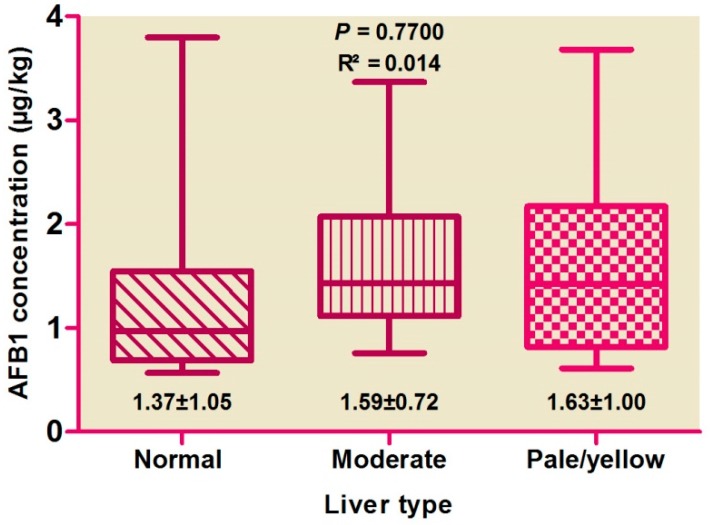
Distribution of aflatoxin B1 concentration between liver types.

**Figure 2:**
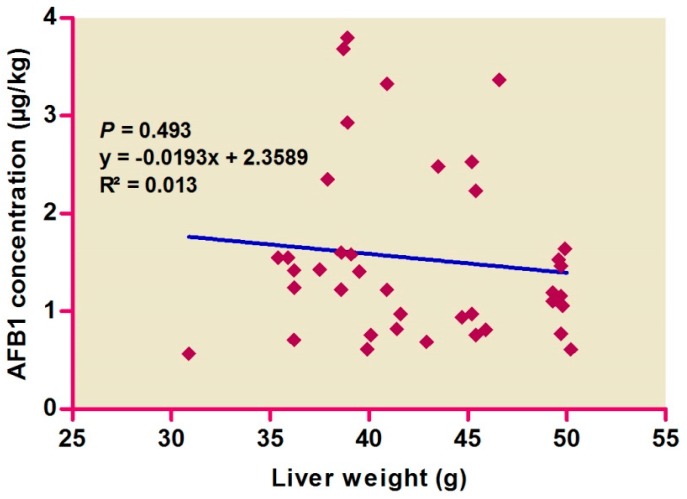
Relationship between aflatoxin B1 concentration in liver samples and the liver weights.

Correlations among hepatic AFB1 levels and colors (Figure 1) and weights (Figure 2) were evaluated, with no difference found by color score (*p* > 0.05), suggesting the absence of any correlation. AFB1 levels were found to be high in pale livers, followed by moderate and normal livers.

## 4. Discussion

Aflatoxin contamination in food and foodstuffs represents a major threat to the health of exposed people. AFB1 was detected in chicken liver and gizzard samples from Maputo, thus confirming the poultry’s exposure through feed or feed ingredients, according to previous reports [32,33,34]. AFB1 is known as the most toxic and carcinogenic natural toxicant [10,11,12], which may cause aflatoxicosis and/or induce liver cancer [8,10,14,17,18], as well as, originate metabolite compounds with similar toxic properties, such as aflatoxin [5,10,14]. This emphasizes the importance of monitoring aflatoxins and their metabolites in poultry products.

Overall, the relative high frequency of contamination and AFB1 levels in livers samples from local sector producers—proportionally twice as many as in chicken livers from industrial sector producers—may be explained by the fact that feeding practices and poultry feeds can be major source of aflatoxins [7,31,39,40]. It is a pre-requisite practice in the industrial poultry production sector to ensure adequate conditions and controlled practices of food management and feed storage; regulatory practices and enforcement of proper feed storage guidelines are not as stringent in the local (smallholder family) poultry production sector [1,39,40,41].

Iqbal et al. [7] from Pakistan, using reverse phase High Performance Liquid Chromatography (HPLC) with fluorescence detention, documented that 35% of chicken meat samples were positive for aflatoxins, with the maximum level of AFB1 and total aflatoxins found in the livers 2.98 ± 0.76 and 3.23 ± 0.82 μg/kg, respectively. El-Desouky et al. [5] from Egypt, using immunoaffinity column with HPLC, reported the presence of AFB1 in 45, 32, and 25% of 60 chicken livers, gizzards, and hearts in their study samples, with an overall maximum level of 2.24 μg/kg.

Markov et al. [28] from Croatia reported that mycotoxins were detected in 64% of 90 meat samples analyzed, and found that 10% of the samples were contaminated with AFB1, with a maximum AFB1 level of 3.0 mg/kg. Using different testing systems, Herzallah [27] found levels of total aflatoxins in imported and fresh meat samples collected during March ranged from 0.15 to 6.36 μg/kg.

In a review study, Rodriguez-Amaya and Sabino [42] from Brazil found variable frequency of AFB1 contamination in chicken liver samples; with positivity at ~50% of samples tested, and maximum mean level 3.2 μg/kg. In a separate Brazilian investigation, Stamford et al. [43], using ELISA and Thin Layer Chromatography (TLC), found AFB1 concentrations in livers samples from different slaughterhouses ranging from 0.54 to 2.41 μg/kg. Most of these previous findings are in complete agreement with the findings of the present study, although with different tissues and species.

Therefore, it may be noted that unlike the present study, most of the cited reports used HPLC and TLC for aflatoxin content determination. This methodological approach is due to the fact that these techniques are conventional and validated, in addition to their high efficiency, high sensitivity, and high resolution, with low detection limit (about 0.1 ng/kg) [44,45]. However, due to the special requirements, expensive apparatus and instruments, as well as laborious and time consuming preparation of samples of the conventional methods [44,45], immunoassay methods such as ELISA have been frequently used recently—especially in low income countries—for mycotoxin examination in food and agricultural products [44,45,46]. According to Zheng et al. [45], ELISA test kits are favored as high throughput assays with low sample volume requirements and often less sample extract clean-up procedures compared to conventional methods. Moreover, the method is rapid, simple, specific, sensitive, and portable for use in the field, in addition to being fully quantitative [45,46]. Although, it is also reported that clean-up by IAC prior to ELISA testing is needed [7,44,45]; a step which was not performed in the present study due to financial limitations. Many commercial ELISA aflatoxin tests, such as that used in this study, often without purification, only need the defatting step prior to analysis, which makes the test essentially useful as a screening test for routine quality control of foodstuffs contamination [45,46]. Bahobail et al. [46] using an ELISA MaxSignal^®^ commercial kit (MaxSignal^®^, Bioo Scientific Corporation, USA)—without prior cleanup procedure—found trace amounts of total aflatoxin contamination in egg samples (ranging from <1 to 1.19 μg/kg).

In poultry animals, mycotoxicosis can present direct or acute symptoms, including negative effects on their immune system, but reductions are less obvious [2,14,16,20,47]. Among the symptoms, gross hepatic changes in color, volume or weight, and consistency are frequently the first and most observed in poultry species [16,43,47,48,49,50]. These parameters, in addition to odor, are commonly used as selection criteria in several countries to approve livers and other edible viscera for in natura commercialization [37,43]. In such cases, the findings of the liver color assessment are considered as primary effects of aflatoxicosis in the current study would have led to condemnation of approximately 53% of the total sampled livers (“moderate” or “pale”). However, similar hepatic changes can be observed as result of other factors such as pre-harvest feed withdrawal [51] and exposure of chickens to high environmental temperatures [52], hence only 39% of total livers showed AFB1 levels.

The low AFB1 levels, as well as their non-correlation with liver morphological findings in the present study, can be hypothesized as a result of the natural variation of feed contamination and the consequent exposure of the chickens. Although feed levels were not quantified in this study, the effects of aflatoxins on animals vary depending on the concentration and duration of consumption, breed, and diet [26]. The detection of AFB1 in livers and gizzards, as well as other of animal origin products, occurred when diets were contaminated with AFB1 levels between 2.5 to 20 mg/kg [26], with increases of AFB1 in the diet resulting in higher residue levels in animal tissues [19,21,29,53,54].

Wolzak et al. [55] reported that tissue residues of aflatoxin were highest in the liver, gizzard, and kidney when boilers were exposed for four weeks to a mixture of AFB1 and AFB2. After seven days of removal of the contaminated feed, aflatoxin could not be detected in aforementioned tissues. In this regard, Hussain et al. [56] recently reported that during long exposures to AFB1, the elimination of AFB1 in chicken increases. The authors also concluded that despite the increasing AFB1 residues in chicken tissues as a result of an increase in dietary concentration, contamination decreases with the increasing age of chickens. It has also been reported that concomitant exposure of birds to multiple types of mycotoxins may increase the excretion of toxins, thereby reducing their retention [56].

The current low AFB1 levels recorded in poultry tissues in this study (0.57–3.8 µg/kg) suggest improvements in feed handling and feed safety over the past decade. Nonetheless, continued vigilance is necessary to monitor efficacy and progress. Since any improper production, feed handling, or storage may result in the development of toxigenic fungi and the consequent production of aflatoxins, it is relevant to conduct a regular screening for aflatoxins and other mycotoxins in poultry feeds, as well as in meat products to minimize both animal and human health hazards.

## 5. Conclusions

The AFB1 contamination values measured in chicken livers and gizzards were lower than the current allowable limit in Mozambique, suggesting that these products may pose minimal risk to consumer health. Since the consumption of chicken meat, including giblets (livers and gizzards), is increasing due to its availability at reasonable prices, the widespread findings of AFB1 (up to 67% in LS produced livers) present alarming baseline information with human health implications, as well as national economic factors associated with poultry production.

The use of a relatively rapid and economic ELISA assay technique may provide a useful analytical tool for developing better standards of monitoring (and, ultimately, eliminating or controlling) the presence of these potential toxins in feed and animal source food in particular chicken meat.

Nevertheless, due to the limitation of the ELISA method, as well as the reduced period of sampling, there is a need, before determination, to include an immunoaffinity cleanup step or consider the test validation, and also to increase the sampling period, to guarantee accurate quantitative measurements and inclusion of the several influencing factors. In addition, permissible limits should be defined and implemented for feeds to avoid fungal contamination; although not a focus of this current study, the ELISA test may also be applicable for feedstuff evaluation as part of overall food safety programs in Mozambique.

## Figures and Tables

**Table 1:** Morphological evaluation of liver samples.

Liver Type	IS Livers				LS Livers			
	Samples	Weight (g)			Samples	Weight (g)		
		Range	Mean ± SD	95% CI		Range	Mean ± SD	95% CI
Normal	35 (50.0)	23.80–46.80	35.47 ± 0.79 *^a^*	33.85–37.10	12 (40.0)	30.20–45.60	39.24 ± 1.40 *^a^*	36.16–42.32
Moderate	28 (40.0)	30.20–45.40	39.28 ± 0.78 *^a^*	37.66–40.89	12 (40.0)	40.80–49.70	46.03 ± 0.90 *^a^*	44.03–48.02
Pale/yellow	7 (10.0)	37.90–50.20	45.59 ± 2.06 *^a^*	40.54–50.63	6 (20.0)	36.20–49.70	41.67 ± 2.23 *^a^*	35.93–47.31
Total	70 (70.0)	23.80–50.20	38.00 ± 5.48 *^b^*	36.70–39.30	30 (30.0)	30.20–49.70	42.40 ± 5.24 *^b^*	40.50–44.40

IS: Industrial sector or formal producers; LS: Local sector or informal producers; SD: Standard deviation; CI: Confidence interval of mean; The data in parentheses represents the percentage (%); *^a^* The values differ statistically (*p* < 0.05) by the *t* student test; *^b^* The values differ statistically (*p* < 0.05) by the ANOVA test.

**Table 2:** Recovery of aflatoxin B_1_ in artificially contaminated (spiked) chicken liver.

AFB1 Spiked Level (µg/kg)	AFB1 Recovery * (µg/kg)						R (%)
	Assay 1	Assay 2	Assay 3	Assay 4	Assay 5	Mean ± SD	
5.0	4.67	4.64	4.34	4.54	4.49	4.54 ± 0.13	90.20
10.0	9.23	9.54	9.17	9.34	9.29	9.31 ± 0.14	93.14

SD: Standard deviation; R (%): Recovery rate; * All results are from the same two negative liver samples of a preliminary testing.

**Table 3:** Frequency of contamination and aflatoxin B_1_ concentrations in chicken livers and gizzards.

Sample Type	Samples		AFB1 Content (µg/kg)		Samples with Level	
	Analyzed	Positive	Range	Mean ± SD	<10 * µg/kg	>10 µg/kg
IS livers	70	19 (27.1) *^a^*	0.61–2.48	1.35 ± 0.58 *^b^*	70	0
LS livers	30	20 (66.7) *^a^*	0.57–3.80	1.73 ± 1.09 *^b^*	30	0
Total	100	39 (39.0)	0.57–3.80	1.54 ± 0.89 *^b^*	100	0
IS gizzards	50	2 (4.0) *^a^*	0.81–1.34	1.07 ± 0.37 *^b^*	50	0
LS gizzards	30	9 (30.0) *^a^*	0.68–2.12	1.04 ± 0.44 *^b^*	30	0
Total	80	11 (13.8)	0.68–2.12	1.06 ± 0.42 *^b^*	80	0

IS: Industrial sector or formal producers; LS: Local sector or informal producers; SD: Standard deviation; The data in parentheses represents the percentage (%) of positive samples; * Maximum tolerated level from the Codex Alimentarius regulations for human food [6,24,35]; *^a^* The values differ statistically (*p* < 0.05) by the Chi-square and fisher tests; *^b^* The values do not differ statistically (*p* > 0.05) by the ANOVA test.
